# Ethyl lauroyl arginate: An update on the antimicrobial potential and application in the food systems: a review

**DOI:** 10.3389/fmicb.2023.1125808

**Published:** 2023-02-23

**Authors:** Yunfang Ma, Yanqing Ma, Lei Chi, Shaodan Wang, Dianhe Zhang, Qisen Xiang

**Affiliations:** ^1^College of Food and Bioengineering, Zhengzhou University of Light Industry, Zhengzhou, China; ^2^Henan Key Laboratory of Cold Chain Food Quality and Safety Control, Zhengzhou, China

**Keywords:** ethyl lauroyl arginate, antimicrobial efficacy, decontamination, food, mechanism

## Abstract

Ethyl lauroyl arginate (ELA), a cationic surfactant with low toxicity, displays excellent antimicrobial activity against a broad range of microorganisms. ELA has been approved as generally recognized as safe (GRAS) for widespread application in certain foods at a maximum concentration of 200 ppm. In this context, extensive research has been carried out on the application of ELA in food preservation for improving the microbiological safety and quality characteristics of various food products. This study aims to present a general review of recent research progress on the antimicrobial efficacy of ELA and its application in the food industry. It covers the physicochemical properties, antimicrobial efficacy of ELA, and the underlying mechanism of its action. This review also summarizes the application of ELA in various foods products as well as its influence on the nutritional and sensory properties of such foods. Additionally, the main factors influencing the antimicrobial efficacy of ELA are reviewed in this work, and combination strategies are provided to enhance the antimicrobial potency of ELA. Finally, the concluding remarks and possible recommendations for the future research are also presented in this review. In summary, ELA has the great potential application in the food industry. Overall, the present review intends to improve the application of ELA in food preservation.

## Introduction

Food safety is a highly complex public health issue in the world, which leads to serious adverse health consequences and large economic losses. Among various contaminants, microorganisms are reported as an important factor affecting the safety of food products. Fresh produce and processed foods can be easily contaminated by various microorganisms including bacteria, molds, yeasts, and viruses across the whole food supply chain. Bacteria and viruses are the most common cause of food poisoning and can result in a myriad of symptoms, ranging from diarrhea syndromes, fever, and even death (Bintsis, 2017). The United States Department of Agriculture (USDA) estimated that 15 major pathogens (such as *Campylobacter* spp., *Clostridium perfringens*, *Salmonella*, and norovirus) led to roughly 8.9 million cases of illness and an economic cost of around 17.6 billion dollars in 2018 in the United States (United States Department of Agriculture, Economic Research Service, 2022). Some microorganisms can also cause food spoilage, resulting in deterioration of the nutritional and sensory properties of foods as well as significant economic losses (Ishangulyyev et al., 2019). Therefore, how to ensure the safety of food products is one of the most important issues in the food industry. The use of chemical antimicrobial agents is one of the best and the most effective methods of preserving foods. Food antimicrobial agents can inhibit the growth of or inactivate various spoilage and pathogenic microorganisms, thereby extending the shelf life of food products. Various chemical preservatives have been used commonly so far in the food industry, such as organic acids and their salts, nitrates, nitrites, and sulfur dioxide. However, an increasing number of studies suggest that long-term exposure to chemical antimicrobial agents may result in potential health risks (Javanmardi et al., 2019; Karwowska and Kononiuk, 2020), which has received high attention both from consumers and manufacturers. Therefore, numerous efforts have been made to develop alternative antimicrobial compounds with higher safety and efficacy.

Ethyl lauroyl arginate (ELA), also known as ethyl-N^α^-lauroyl-Larginate, is an amino acid-based cationic surfactant, which is synthesized from L-arginine, lauric acid, and ethanol. ELA is considered one of the most potent antimicrobial substances among novel food additives for its broad-spectrum antimicrobial activity against a wide range of bacteria, yeasts, and filamentous fungi (Ma et al., 2020; Demircan and Özdestan Ocak, 2021). The use of ELA as a food preservative has been approved by the Food Drug Administration (FDA), the European Food Safety Agency (EFSA), and other countries. In this context, the application of ELA in various food products has been widely investigated in recent years. It is therefore important to develop a better understanding of the application of ELA in the food industry. So this article aims to provide a general overview of the physicochemical properties and antimicrobial activity of ELA as well as its underlying mechanism of action. Moreover, this review also summarizes the use of ELA to improve the microbiological safety, quality attributes, and shelf life of various food products such as fruit and vegetables, meat, poultry, and dairy products. Furthermore, a detailed comprehensive review is performed on the combination strategies to enhance the potency of ELA and the factors influencing its antimicrobial efficacy. Finally, concluding remarks and suggestions for further work are also presented.

## Basis of ELA

### Properties and synthesis of ELA

**Figure 1:**
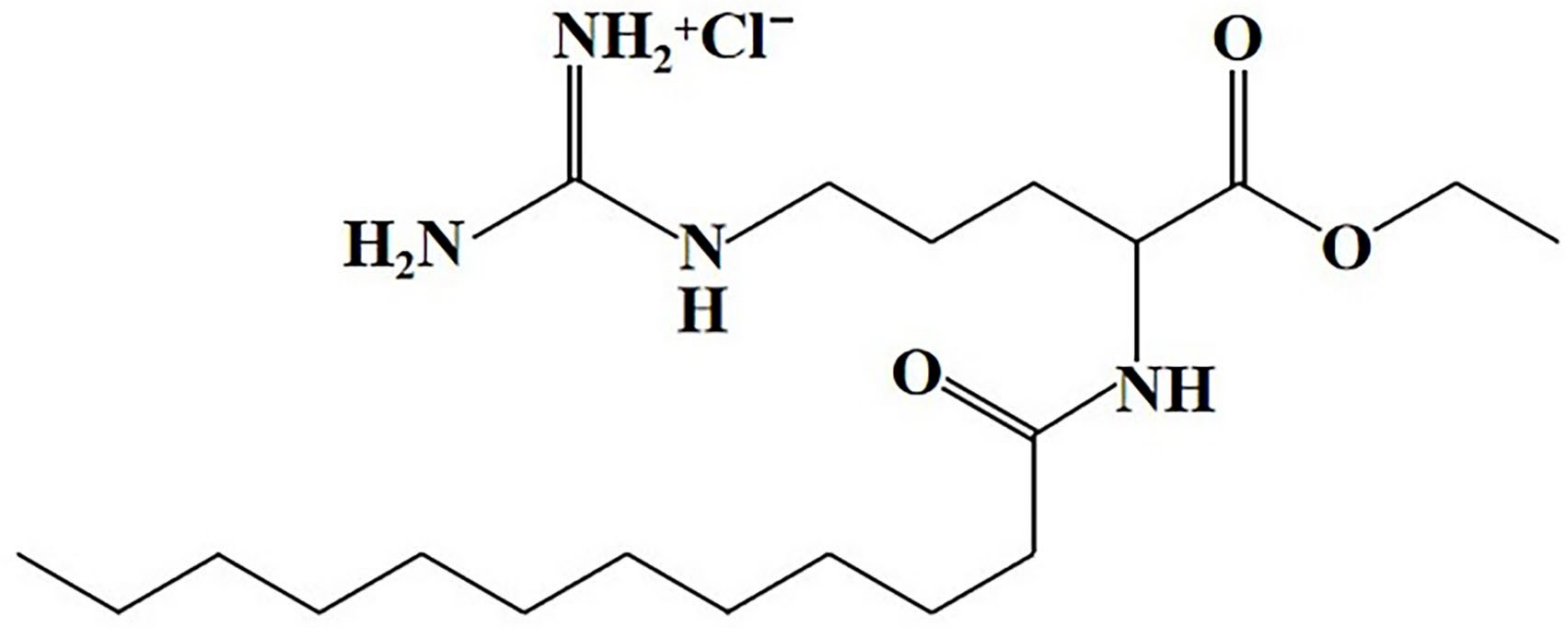
Chemical structure of ELA.

Ethyl lauroyl arginate hydrochloride (C_20_H_40_N_4_O_3_HCl, CAS NO. 60372–77-2) is a white hygroscopic powder with a melting point at 50.5°C to 58°C. ELA has a molecular weight of 421.023 g/mol and has good water solubility (greater than 247 g/kg at 20°Cl; EFSA, 2007). The pKa of ELA is at about 10–11 and the isoelectric point is above pH 12 (Czakaj et al., 2021). The structural formula is presented in Figure 1. As a surfactant, ELA exhibits self-assembly behavior and forms micelles above the critical micelle concentration (CMC). The CMC of ELA is determined to be 0.18–0.21% (w/v; Asker et al., 2008; Bai et al., 2018). ELA includes a wide range of surface tension values from 25.4 to 31.8 (Infante et al., 1984; Czakaj et al., 2021). ELA is also used to prepare oil-in-water (O/W) emulsion with a hydrophilic–lipophilic balance (HLB) value of 10.5 (Ma et al., 2020). ELA is stable for more than 2 years at room temperature when protected in a closed container. The half-life of ELA is greater than 1 year at pH 4, 57 d at pH 7, and 34 h at pH 9 during 25°C storage, indicating that the hydrolysis of ELA is accelerated in alkaline conditions (EFSA, 2007). However, ELA has a bitter taste above 50 ppm in food and beverage products (Zheng, 2014), which may adversely influence the taste and flavor of food products.

In 1984, ELA was first synthesized by the Higher Council of Scientific Research (CSIC) in Barcelona. It was then patented and commercialized by the Venta de Especialidades Químicas S.A. (VEDEQSA) company of LAMIRSA GROUP. ELA can be synthesized by the esterification reaction between L-arginine and ethanol, followed by the amidation reaction between the obtained ethyl arginine and lauroyl chloride in an aqueous medium under appropriate temperature (10–15°C) and pH conditions (6.7–6.9). After filtration and drying, the resultant ELA is recovered as the hydrochloride salt (EFSA, 2019).

### Metabolism and toxicity of ELA

**Figure 2:**
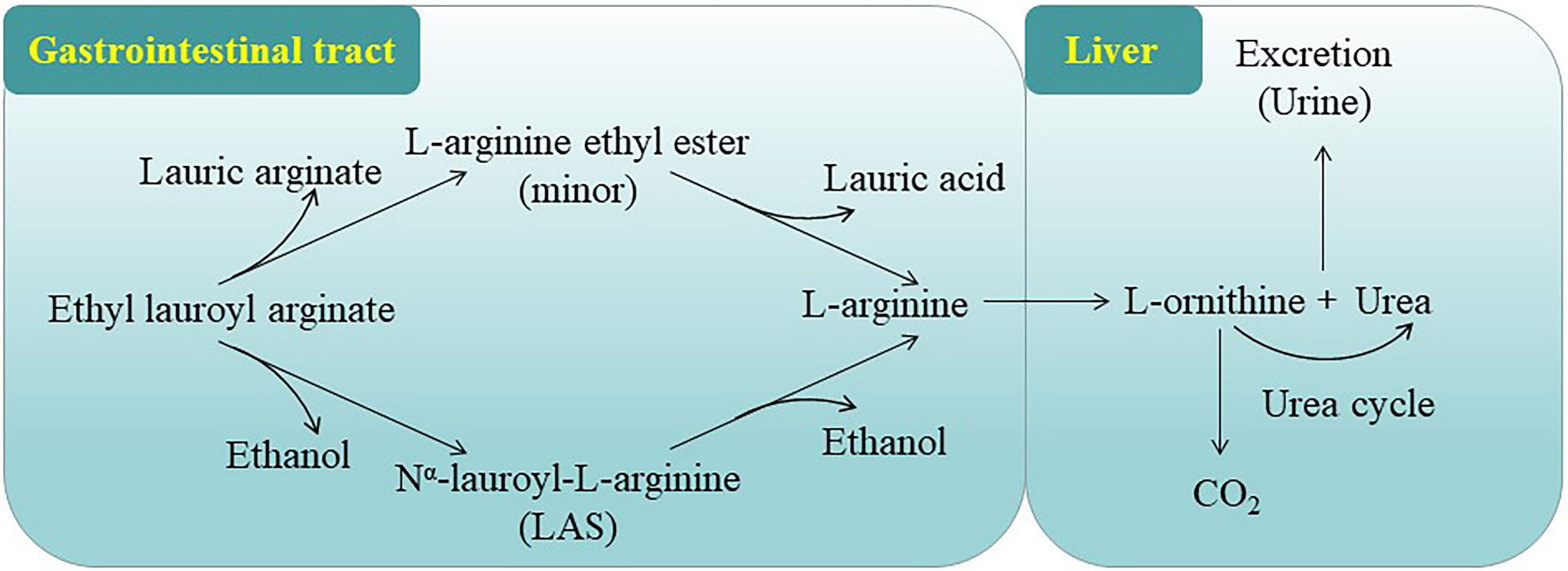
Proposed metabolic pathway of ELA (EFSA, 2007; Hawkins et al., 2009).

According to the *in vivo* and *in vitro* studies, ELA is rapidly converted to L-arginine ethyl ester *via* the cleavage of lauroyl side chain or N^α^-lauroyl-L-arginine (LAS) *via* the loss of ethyl ester (Figure 2). The resulting intermediates are further hydrolyzed to form L-arginine, which is further metabolized to urea and ornithine. Ornithine is further converted to CO_2_ and urea. Lauric acid is a saturated fat widely found in many vegetable fats and can enter normal fatty acid metabolism. Alcohol can be degraded to CO_2_ and water *via* some normal metabolic processes (EFSA, 2007; Hawkins et al., 2009). These data suggest that ELA is primarily and rapidly metabolized *in vivo*.

The potential toxicity of ELA has been well investigated. Based on two short-term toxicity studies, ELA has no effects on the white blood cells parameters of rats (EFSA, 2019). It has reported that highest dose ELA (15,000 mg/kg feed) had noted effects on the delay (average of 4 days) in vaginal opening in the female offspring (EFSA, 2019). However, the observed delay in vaginal opening is of no long-term toxicological relevance. The research data suggest that ELA has very low mammalian toxicity (Ruckman et al., 2004; EFSA, 2007). More toxicological metabolic investigations are reviewed by Ruckman et al. (2004). The lowest No Observed Adverse Effect Levels (NOAELs) of ELA were 47 and 56 mg/kg body weight (bw) per day for males and females, respectively (EFSA, 2019). The EFSA panel established an acceptable daily intake (ADI) of 0.5 mg/kg bw for ELA (EFSA, 2007). In June 2008, the Joint FAO/WHO Expert Committee on Food Additives (JECFA) established an ADI of 4 mg/kg bw for ethyl-N^α^-lauroyl-l-arginate, the active ingredient of ELA (EFSA, 2019).

### Legal aspects of ELA use in foods

In September 2005, FDA presented the No Objection Letter for ELA to be a generally recognized as safe (GRAS) compound and used as an antimicrobial agent in different types of foods at levels up to 200 ppm (FDA, 2005). In addition, the USDA approved the use of ELA in meat and poultry products at up to 200 ppm (United States Department of Agriculture, Food Safety and Inspection Service, 2022). In the European Union, ELA was evaluated for safety by the EFSA in April 2007 at the 39th EFSA evaluated Codex Committee on Food Additives and Contaminants (CCFAC). EFSA assigned the E 243 number for ELA in 2013. In May 2014, the Commission Regulation (EU) No 506/2014 was published, authoring the use of ELA as a preservative in certain heat-treated meat products. In August 2014, ELA was approved in Canada as a preservative in various foods. At present, ELA is currently authorized as a food preservative in other countries, such as Australia, New Zealand, Mexico, Colombia, Chile, Israel and Turkey, United Arab Emirates, and Vietnam, at a maximum concentration of 200 ppm (EFSA, 2019; Motta et al., 2020).

## Antimicrobial activity and mechanism of ELA

ELA has attracted increasing interest due to its better antimicrobial activity against bacteria, yeasts, and molds.

### Antibacterial and antibiofilm activity of ELA

Previous work had shown that ELA is active against various food spoilage and pathogenic bacteria. Table 1 summarizes the minimum inhibitory concentrations (MICs) and minimum bactericidal concentrations (MBCs) of ELA against different bacteria. The diverse MIC and MBC values of ELA are observed in the literature (Table 1) as a consequence of differences in the strains and serotypes of bacteria tested, the methods used, medium composition, and so on. According to Table 1, Gram-negative bacteria tend to be more resistant to ELA than Gram-positive ones (Infante et al., 1984; Rodríguez et al., 2004; Becerril et al., 2013; Suksathit and Tangwatcharin, 2013). Gram-negative bacteria are surrounded by an external membrane primarily composed of lipopolysaccharides and phospholipids, which acts as a permeability barrier against external toxic compounds (Miller, 2016). Gram-positive bacteria are more sensitive to ELA due to lacking the additional protection afforded by the outer membrane.

**Table 1:** MICs and MBCs values of ELA against bacteria.

Bacteria	Gram type	Medium	MIC (μg/ml)	MBC (μg/ml)	Reference
*S*. *aureus* ATCC6538	Positive	MHB	8	–	Rodríguez et al. (2004)
*S*. Typhimurium ATCC 14028	Negative	MHB	32	–	Rodríguez et al. (2004)
*L*. *monocytogenes* (21 strains)	Positive	TSB	25	–	Soni et al. (2010)
*S*. *aureus* ATCC 29213	Positive	TSA	12.5	50	Becerril et al. (2013)
*L*. *innocua* DSMZ 20649	Positive	TSA	25	25	Becerril et al. (2013)
*E*. *coli* ATCC 25922	Negative	TSA	25	25	Becerril et al. (2013)
*P*. *aeruginosa* ATCC 27853	Negative	TSA	100	100	Becerril et al. (2013)
*S*. *enterica* CECT556	Negative	TSA	25	25	Becerril et al. (2013)
*L*. *monocytogenes* CECT934	Positive	TSA, TSB	8	16	Higueras et al. (2013)
*S*. *aureus* MIM178	Positive	TSA, TSB	8	16	Higueras et al. (2013)
*E*. *coli* CECT434	Negative	TSA, TSB	16	24	Higueras et al. (2013)
*P*. *putida* ATCC12633	Negative	TSA, TSB	16	24	Higueras et al. (2013)
*S*. *enterica* CECT4300	Negative	TSA, TSB	16	24	Higueras et al. (2013)
*L*. *monocytogenes* Scott A	Positive	TSB	11.8	23.5	Ma et al. (2013)
*E*. *coli* O157:H7 ATCC 43895	Negative	TSB	11.8	11.8	Ma et al. (2013)
*S*. Enteritidis	Negative	TSB	23.5	23.5	Ma et al. (2013)
*L*. *monocytogenes* TSULM1	Positive	MHB	8	32	Suksathit and Tangwatcharin (2013)
*S*. Rissen TSUSR1	Negative	MHB	16	32	Suksathit and Tangwatcharin (2013)
*E*. *coli* O157:H7 CECT 5947	Negative	–	25	25	Otero et al. (2014)
*E*. *coli* O157:H7 M364VO	Negative	–	25	25	Otero et al. (2014)
*L*. *plantarum* ATCC 8014	Positive	MHB	32	–	Coronel-León et al. (2016)
*Y*. *enterocolitica* ATCC 9610	Negative	MHB	8	–	Coronel-León et al. (2016)
*E*. *coli* O157:H7 B6-914	Negative	TSBYE	20	–	Fu et al. (2017a)
*B*. *cereus* DSM 31	Positive	MHB	16	–	Nübling et al. (2017b)
*S*. *aureus* DSM 20231	Positive	MHB	4	–	Nübling et al. (2017b)
*L*. *monocytogenes* DSM 20600	Positive	MHB	16	–	Nübling et al. (2017b)
*P*. *aeruginosa* DSM 1117	Negative	MHB	32	–	Nübling et al. (2017b)
*S*. *enterica* Typhimurium DSM 17058	Negative	MHB	16	–	Nübling et al. (2017b)
*L*. *monocytogenes* 19,113	Positive	TSB	10.0	12.0	Sadekuzzaman et al. (2017)
*E*. *coli* O157:H7 NCCP 11090	Negative	TSB	18.3	20.0	Sadekuzzaman et al. (2017)
*S*. Enteritidis ATCC13076	Negative	TSB	11.0	12.5	Sadekuzzaman et al. (2017)
*S*. Typhimurium ATCC14028	Negative	TSB	11.0	12.5	Sadekuzzaman et al. (2017)
*P*. *carotovorum* subsp. *carotovorum* CGMCC1.3614	Negative	TSB	25	–	Li et al. (2018)

MHB, Muller Hinton Broth; TSA, Tryptic Soy Agar; TSB, Tryptic Soy Broth; TSBYE, Tryptic Soy Broth with 0.6% Yeast Extract.

Biofilms are defined as microbial communities attached to a surface and encased in a matrix of extracellular polymeric secretions. Cells in a biofilm demonstrate much greater resistance to several acute environmental stressors (Galié et al., 2018). After treatment with ELA (50, 100, and 200 μg/ml) for 2 h, *L*. *monocytogenes*, *S*. Enteritidis and *S*. Typhimurium in biofilms on stainless steel and rubber surfaces were reduced by up to 7 and 3.5 log_10_ CFU/cm^2^, respectively (Sadekuzzaman et al., 2017). Similar results were obtained by Fu et al. (2017a) and Fernández et al. (2018). In addition, Kim et al. (2017) speculated that the antibiofilm activity of ELA against *Pseudomonas aeruginosa* might be attributed to its iron chelation activity and blocking effect on the iron signals associated with the biofilm development.

### Antifungal activity of ELA

Yeasts and molds can cause various degrees of deterioration and decomposition of foods, such as grains, nuts, meat, milk, fruits, and vegetables. According to literature, ELA displays strong antifungal activities against various yeasts and molds (Table 2). Typically, the MIC values of ELA against *Saccharomyces cerevisiae*, *Candida albicans*, and *Zygosaccharomyces bailii* were 35, 112.5, and 62.5 μg/ml, respectively (Loeffler et al., 2014). Similarly, ELA showed *in vitro* antifungal potential against *Botrytis cinerea*, *Alternaria alternate*, *Penicillium italicum*, and *Penicillium digitatum* with a MIC value of 400, 200, 400, and 400 μg/ml, respectively (Li et al., 2018). It should be pointed out, however, that yeasts and molds exhibit significantly greater resistance to ELA than bacterial cells based upon the MIC values in Tables 1, 2, which may be due to the differences in the structure and chemical composition of cell walls.

**Table 2:** MICs and MBCs values of ELA against yeasts and fungi.

Microorganisms	Medium	MIC (μg/ml)	MBC (μg/ml)	Reference
*C*. *utilis* CCY29.38.1	TSB	16	24	Higueras et al. (2013)
*Candida utilis* CCY29.38.1	MEA	104	120	Higueras et al. (2013)
*S*. *cerevisiae* NCYC2959	TSB	16	24	Higueras et al. (2013)
*S*. *cerevisiae* NCYC2959	MEA	104	120	Higueras et al. (2013)
*T*. *pinus* IMAP4543	TSB	4	8	Higueras et al. (2013)
*T*. *pinus* IMAP4543	MEA	32	48	Higueras et al. (2013)
*S*. *cerevisiae* LTH 6759	SDB	20	35	Loeffler et al. (2014)
*C*. *albicans* LTH 6758	SDB	50	112.5	Loeffler et al. (2014)
*Z*. *bailii* LTH 67457	SDB	30	62.5	Loeffler et al. (2014)
*A*. *niger* MIM 28	MEA	24	320	Higueras et al. (2013)
*C*. *cladosporioides* MIM259	MEA	24	80	Higueras et al. (2013)
*P*. *chrysogenum* MIM29	MEA	120	280	Higueras et al. (2013)
*B*. *cinerea* CGMCC3.4584	PDA	400	–	Li et al. (2018)
*A*. *alternate* CGMCC3.7809	PDA	200	–	Li et al. (2018)
*P*. *italicum* CGMCC3.8284	PDA	400	–	Li et al. (2018)
*P*. *digitatum* CGMCC3.7771	PDA	400	–	Li et al. (2018)

MEA, Malt Extract Agar; PDA, Potato Dextrose Agar; SDB, Sabouraud Dextrose Broth; TSB, Tryptic Soy Broth.

### Possible antimicrobial mechanism of ELA

**Figure 3:**
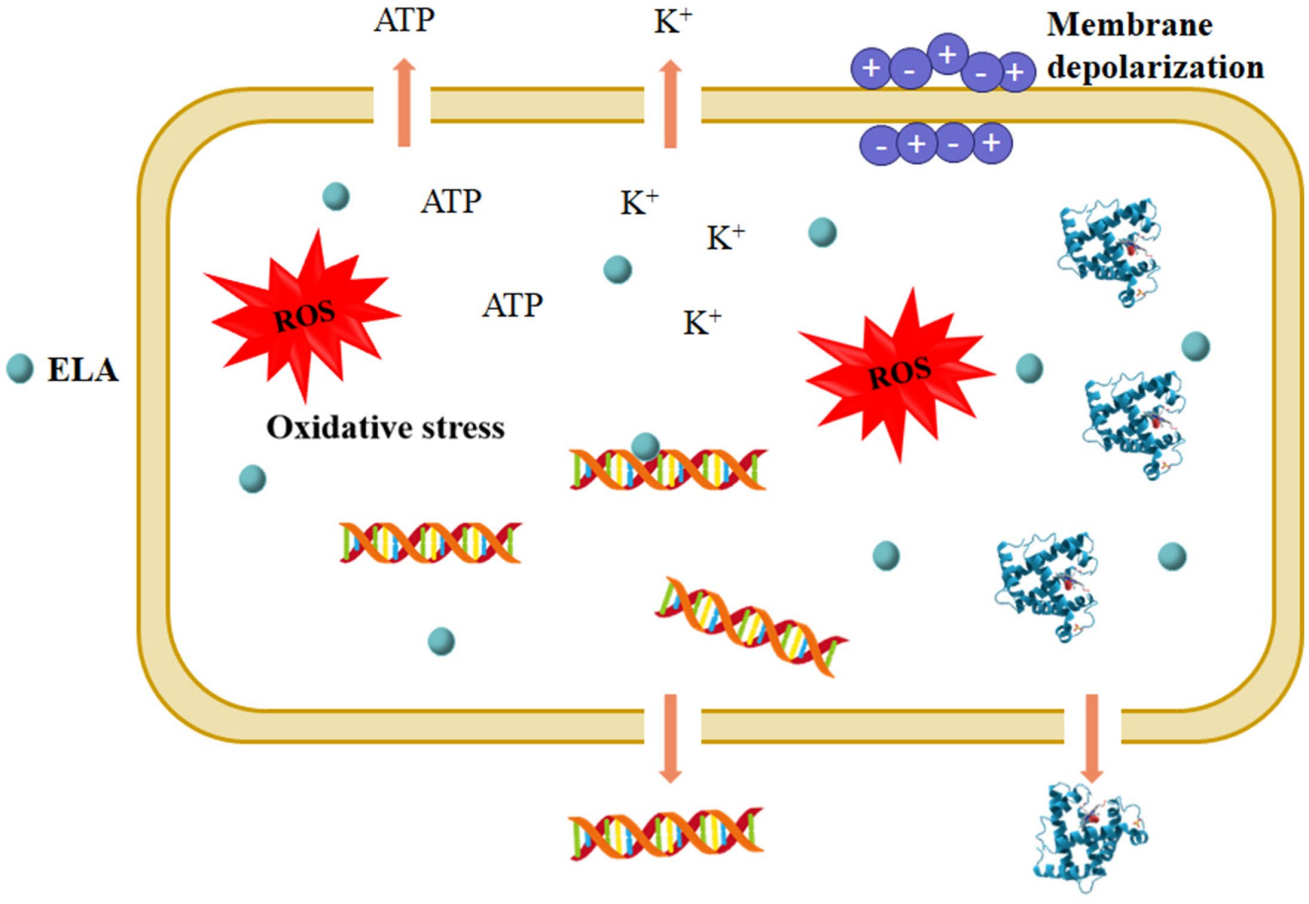
Proposed mechanisms underlying the antimicrobial action of ELA. ROS, reactive oxygen species.

Although the antimicrobial mechanism of ELA has not been fully deciphered, microbial cell membranes are thought to be the main target of ELA. As a cationic surfactant, ELA can damage cell membranes, leading to the disruption of cell membranes, the lose of membrane potential, and leakage of cellular components (Figure 3). Pattanayaiying et al. (2014) investigated the effects of ELA on the morphological and ultrastructural features of *E*. *coli* O157:H7, *L*. *monocytogenes*, and *Brochothrix thermosphacta* cells by scanning electron microscopy and transmission electron microscopy. The results showed that ELA caused remarkable changes in the ultrastructure and morphology of bacterial cells, such as distorted and dimpled *E*. *coli* O157: H7 cells and the intracytoplasmic coagulation of *B*. *thermosphacta* cells. In another work, Coronel-León et al. (2016) studied the influences of ELA on the membrane potential and permeability of bacterial cells by flow cytometry. After ELA treatment at 1 × MIC, the percentage of bisoxonol-positive *Yersinia enterocolitica* and *Lactobacillus plantarum* cells were increased to 1.8 and 0.3%, respectively, significantly higher than that of the control cells (0.6 and 0.1%, respectively), suggesting the depolarization of the membrane. The percentages of propidium iodide-positive *Y*. *enterocolitica* and *L*. *plantarum* cells increased to 97.8 and 99.6%, respectively, significantly higher than that of the control cells (0.7 and 0.01%, respectively), after exposure to ELA at 1 × MIC, indicating the significant increase in cell membrane permeability (Coronel-León et al., 2016). As results of cell membrane disruption, ELA causes releases of intracellular contents such as potassium (Rodríguez et al., 2004; Coronel-León et al., 2016), proteins (Xu et al., 2018; Zhao et al., 2022a), nucleic acids (Xu et al., 2018; Yang et al., 2019), and ATP (Ma et al., 2016c), and disrupt normal cellular metabolism, thereby leading to cell death. Meanwhile, ELA may bind to the cellular components with negative charges after entry into the microbial cells, thereby affecting the normal metabolic function. For instance, Ma et al. (2016c) observed a strong interaction between ELA and bacterial DNA, a negatively charged polymer, through electrostatic attraction and hydrophobic interaction.

The generation of intracellular reactive oxygen species (ROS) is thought to be involved in the antimicrobial action of ELA (Figure 3). As revealed by Yang et al. (2019), the co-administration of antioxidants (such as glutathione and ascorbic acid) effectively suppressed the inactivation efficacy of ELA against *E*. *coli* O157:H7 and *L*. *innocua*, suggesting that oxidative stress was directly associated with ELA-induced microbial inactivation. Excessive ROS can cause irreversible oxidative damage to cellular components (such as DNA, proteins, and lipids) and activate signaling pathways, leading to the disruption of normal cellular functions and ultimately cell death. However, the pathways of ELA-induced ROS generation in microbial cells are still not clear, and more detailed research is required.

In summary, the antimicrobial effect of ELA is mainly related to membrane damage and oxidative stress (Figure 3). On the whole, the present research is mainly focused on the influences of ELA on the structure and function of microbial cells. While multiomics-based analyses (i.e., transcriptomics, proteomics, and metabolomics) should be used to elucidate the underlying molecular mechanisms for the action of ELA. Additionally, the molecular dynamics simulation can be used to explore the membrane binding and disruption mechanisms of ELA (Velasco-Bolom et al., 2018).

## Application of ELA as a food preservative

**Figure 4:**
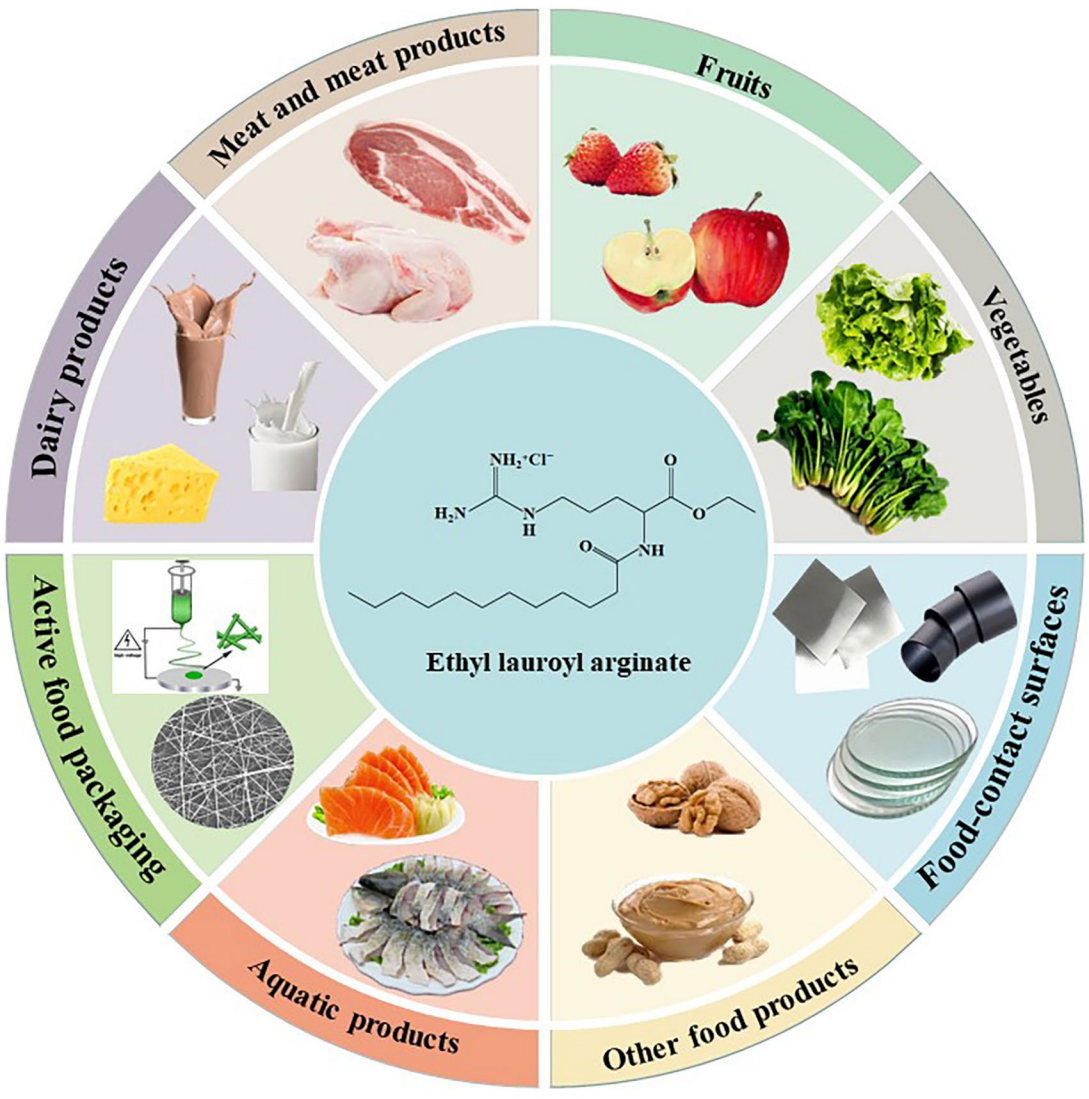
The application of ELA in the food industry.

ELA has been widely studied to extend the microbiological shelf-life and the quality characteristics of various food categories, such as meat and meat products, fruits and vegetables, dairy products, and aquatic products. Similar to other antimicrobial agents, ELA has the possibility of being directly added into the food samples or being incorporated into an active packaging (Figure 4).

### Meat and meat products

Microbial contamination of meat and meat products is a global health issue, which leads to foodborne illnesses and food poisoning (Heredia and García, 2018). As an ingredient on the GRAS list, ELA has been approved by the FDA for use in meat and poultry products up to a maximum level of 200 ppm (FDA, 2005). In Europe, ELA is currently authorized in heat-treated meat products, smoked sausages and liver paste up to the level of 160 mg/kg food (EFSA, 2019).

ELA can be applied to meat and meat products by direct addition, spray, dip, or brush (Table 3). As reported by Peng et al. (2021), the ELA supplement at 0.1 or 0.3 g/kg effectively inhibited the growth of bacteria in yak sausage during 15 days of storage at 0–4°C. Meanwhile, ELA also resulted in significant reductions in drip loss, cooking loss, total volatile basic nitrogen (TVB-N) contents, and pH values of yak sausage during the 15 day storage period. Previous studies showed that ELA-spray treatment can effectively inactivate or prevent the growth of microorganisms on meat and meat products, such as ground beef (Dias-Morse et al., 2014), chicken meat (Hawkins et al., 2016), and ham products (Taormina and Dorsa, 2009a; Lavieri et al., 2014).

**Table 3:** Effects of ELA on microbial inactivation meat and meat products.

Meat product	Microorganism	Treatment condition	Microorganisms reduction	Reference
Hams	*L*. *monocytogenes*	Inoculated samples (7.0 log_10_ CFU/ham) were mixed with ELA solution (5% or 10%) in a bag, and then stored at 4°C for 1 d.	3.3 to 6.5 log	Luchansky et al. (2005)
Frankfurters	*L*. *monocytogenes*	Samples with *L*. *monocytogenes* were added with ELA solution at a finial concentration of 22 ppm, vacuum sealed, and then stored at 6°C for 156 d.	Day 0: > 2 log; Day 156: 0.84 to 1.28 log	Martin et al. (2009)
Hams	*L*. *monocytogenes*	Inoculated samples (7.0 log_10_ CFU/ham) were sprayed with ELA solution (9,090 ppm, 15 to 29 ml), vacuum packed, and stored at 4.4 °C for 48 h.	2.04 to 2.86 log	Taormina and Dorsa (2009a)
Frankfurters	*L*. *monocytogenes*	Inoculated samples (7.13 log_10_ CFU/ham) were sprayed with ELA solution (5,000 ppm, 2 to 3 ml), vacuum packed, and stored at 4.4 °C for 8 d.	Day 0: 1.31 to 1.43 log; Day 8: 0.84 to 1.28 log	Taormina and Dorsa (2009b)
Chicken breast	*Salmonella*	The inoculated breasts were treated with ELA solution (200 or 400 ppm, 1 ml), and stored at 4 °C for 7 d.	Day 0: 0.7 to 1.1 log; Day 7: 0.7 to 0.9 log	Sharma et al. (2013)
Chicken breast	*C. jejuni* and psychrotrophs	The inoculated breasts were treated with ELA (200 or 400 mg/kg), and stored at 4 °C for 7 d.	Day 0: *C. jejuni* decreased by 0.8 to 1.3 log, *psychrotrophs* decreased by 1.3 to 2.3 log; Day 7: *C. jejuni* decreased by 1.2 to 1.5 log; Day 14: *psychrotrophs* decreased by 0.6 log	Nair et al. (2014)
Raw chicken thigh meat	*S*. Typhimurium	The inoculated breasts were sprayed with 5% ELA for 60 s and stored at 4 °C for 3 d	1.28 to 1.92 log	Hawkins et al. (2016)
Chicken breast	*L*. *monocytogenes*	The inoculated breasts were treated with ELA (l00, 200, and 400 mg/kg) and stored at 4 °C for 3 d	0.06 to 0.78 log	Yang et al. (2017)
Ground chicken frame	TVC, *S*. Heidelberg and *C*. *jejuni*	The inoculated frames were submerged in 0.1% ELA solution for 10 s, then stored at 4°C for 24 h	*S*. Heidelberg: 0.7-log; *C*. *jejuni*: 1.2-log; no effect on TVC	Moore et al. (2017)
Minced chicken breast meat	*C. jejuni*	The inoculated sample was mixed with ELA (1,000 or 1,500 ppm) in a bag, then stored at 4°C for 14 d.	Day 1: 0.4 to 1.4 log; day 7: 0.3 to 0.7 log; day 14: 0.4 log	Bechstein et al. (2019)
Portioned chicken breast meat	*C. jejuni*	The inoculated sample was mixed with ELA (400 or 1,000 ppm) in a bag, then stored at 4°C for 14 d.	Day 1: 1.4 to 1.5 log; day 7: 1.4 to 1.5 log; day 14: 1.5 log	Bechstein et al. (2019)
Raw beef and pork (300 g)	*E. coli*	Meat samples inoculated with *E. coli* were vacuum-packaged with ELA-coated film and then stored at 6–7°C for 24 d	Raw beef: 0.96 log reduction at day 24; Pork: 0.52 log reduction at day 24	Tirloni et al. (2021)

TVC, total viable count.

Antimicrobial polymer films loaded with ELA have been also designed and applied to meat and meat products (Theinsathid et al., 2012; Higueras et al., 2013; Guo et al., 2014; Muriel-Galet et al., 2015; Kashiri et al., 2019; Hassan and Cutter, 2020; Tirloni et al., 2021). For instance, Higueras et al. (2013) investigated the antimicrobial activity of chitosan-5% ELA film against mesophiles, psychrophiles, *Pseudomonas* spp., colifoms, lactic acid bacteria on fresh chicken breast fillets during storage at 4°C for up to 8 days. The results showed that chitosan-5% ELA film produced 1.78–5.81 log reduction of the tested bacterial species. Similar findings were shown by Hassan and Cutter (2020) that the composite antimicrobial film containing 0.5, 1.0%, or 2.5% ELA also could control foodborne pathogens associated with muscle foods effectively including raw beef, raw chicken breast, and ready-to-eat turkey breast. Besides, it has been showed that ELA is more effective when combined with other antimicrobials, such as potassium lactate, and sodium diacetate (Martin et al., 2009; Porto-Fett et al., 2010; Stopforth et al., 2010), and bacteriophage (Yang et al., 2017).

### Fruits and vegetables

Fresh fruits and vegetables can be contaminated with harmful microorganisms, which continue to be an important source of foodborne disease outbreaks. ELA has been studied as antimicrobial for treating fresh produce (Table 4), such as apples (Shen et al., 2021), strawberries (Li et al., 2021), cantaloupes (Ma et al., 2016c), bell peppers (Li et al., 2020), lettuce (Huang and Nitin, 2017; Nübling et al., 2017b), and spinach (Ruengvisesh et al., 2015; Zhao et al., 2022b). As reported, the *E*. *coli* O157:H7 and *L*. *innocua* cells on lettuce leaves decreased by more than 3-log after washing with ELA solution (0.1% w/w) for 20 min with shaking at 200 rpm (Huang and Nitin, 2017). Moreover, the quality attributes of fresh produce were maintained well after ELA treatment (Ma et al., 2016c; Huang and Nitin, 2017; Nübling et al., 2017a; Li et al., 2020). In addition, ELA can also effectively inactivate foodborne pathogens in the washing water (Nübling et al., 2017b; Zhao et al., 2022b). For instance, after washing endive in water containing 100 mg/l of ELA at 4 or 45°C for 2 min, the total aerobic mesophilic count in the process water of the pilot plant decreased by 2.7- and more than 4-log, respectively (Nübling et al., 2017b). Therefore, ELA may reduce the chance of cross-contamination while washing fruits and vegetables.

**Table 4:** Effects of ELA on microbial inactivation in fruits and vegetables.

Fresh produce	Microorganism	Treatment condition	Results	Reference
Spinach (10 cm^2^)	*E. coli* O157:H7 (K3999) and *S.* Saintpaul	ELA (1.25 g/l) micelles loaded with eugenol (31.25 mg/l), spraying 1, 2, and 3 sprays (1.0 ml per spray)	2 to 3 log reduction	Ruengvisesh et al. (2015)
Spinach	*E. coli* O157:H7 (K3999) and *S.* Saintpaul	ELA (1.25 g/l) micelles loaded with eugenol (31.25 mg/l), immersing in 20 ml of micelle solution for 2 or 5 min	3 to 4 log reduction	Ruengvisesh et al. (2015)
Whole cantaloupes	Total molds and yeast, *S*. *enterica*, *E*. *coli* O157:H7 and *L*. *monocytogenes*	Chitosan coating with 0.1% ELA, 0.1% EDTA, and 1% cinnamon oil, stored at 21°C for up to 14 days.	Total molds/yeast and *S*. *enterica*: reduced to the detection limit; *E*. *coli* and *L*. *monocytogenes*: > 3 log reduction; delayed the changes of color and firmness of cantaloupes during storage; no noticeable effects on total soluble solids content and weight loss	Ma et al. (2016c)
Lettuce leaves (5 × 5 cm)	*E*. *coli* O157:H7 and *L*. *innocua*	Samples were washed in ELA solution (0.1%, w/w) for 20 min with shaking at 200 rpm	> 2 log reduction; no noticeable effects on color, general appearance, and electrolyte leakage rate; a decrease in firmness	Huang and Nitin (2017).
Red oak leaf lettuce (10 g)	*E*. *coli* and *L*. *monocytogenes*	Each leaf was washed in 100 ml of ELA solution (100 mg/l) for 2 min at 6°C and in 100 ml sterile deionized water for 10 s	*E*. *coli* decreased by 2.6 log, *L*. *monocytogenes* decreased by 0.9 log. No bacterial cells were detected in the process water	Nübling et al. (2017a)
Fresh-cut endive	Total aerobic bacteria	Samples were washed in ELA solution (100 mg/l, 4 or 45°C) for 2 min, packed in PP film bags, and stored at 4°C for up to 9 d	1.0 log reduction at day 0, 1.5 log reduction at day 3. Bacterial cells in the process water decreased by 2.7 to 4 log. Sensory parameters were retained during storage	Nübling et al. (2017b)
Green bell pepper		Samples were immersed in the mixed solution of ELA (700 μg/ml), sodium methylparaben (100 μg/ml) and chitosan (10 mg/ml) for 10 min, then were stored at 25°C for 15 d.	At day 15, the percentage of marketable fruit was increased by 30.4%, the decay index decreased by 23.6%. Ascorbic acid and chlorophyll were retained during storage	Li et al. (2020)
Granny Smith apples	*L*. *monocytogenes* (NRRL B-57618, NRRL-33466, and NRRL B-33053)	The inoculated apples were washed with 80 ppm peracetic acid solution containing ELA (0.01% or 0.05%) at 22°C for 2 min	2.40 to 2.62 log reduction	Shen et al. (2021)
Granny Smith apples	*L*. *monocytogenes* (NRRL B-57618, NRRL-33466, and NRRL B-33053)	The inoculated apples were washed with 80 ppm peracetic acid solution containing 0.05% ELA at 46°C for 30 to 120 s	2.90 to 2.95 log reduction	Shen et al. (2021)
Spinach (3 × 3 cm)	*E. coli* O157:H7 and *L*. *monocytogenes*	Samples were immersed in ELA solution (5.0 mg/ml) for 10 min.	*E. coli* O157:H7 decreased by 1.69 log; *L*. *monocytogenes* decreased by 1.52 log;	Zhao et al. (2022b)

In addition, ELA also could effectively inactivate biofilms formed on fruits and vegetables (Sadekuzzaman et al., 2017; Fu et al., 2017a,b). As reported by Sadekuzzaman et al. (2017), 200 μg/ml of ELA reduced *E*. *coli* O157:H7, *L*. *monocytogenes*, *S*. Enteritidis, and *S*. Typhimurium biofilms up to 1.5 log_10_ CFU/cm^2^ on the lettuce leaf surface (*p* < 0.01). Fu et al. (2017a) also made a similar observation that the viable bacterial of 12 h- and 24 h-old *E*. *coli* O157:H7 biofilm on cantaloupe rind decreased by 1.74- and 1.21-log, respectively, after ELA treatment (2 mg/ml) at 22°C for 5 min.

### Dairy products

Dairy products, such as milk, cheese, and butter, can be contaminated with harmful bacteria, resulting in serious health risk. Soni et al. (2010) reported that the population of *L. monocytogenes* in whole milk or skim milk with initial counts of 4 log_10_ CFU/mL was reduced by approximately 1-log with 200 μg/ml of ELA for 24 h. After treatment with 800 μg/ml of ELA for 24 h, *L. monocytogenes* counts in the samples were reduced to nondetectable levels, and there was no subsequent regrowth of *L*. *monocytogenes* after their extended storage at 4°C for up to 15 d. Similar findings were also revealed in queso fresco cheese (Soni et al., 2010, 2012). For queso fresco cheese treated with 200 or 800 μg/ml of ELA, the *L*. *monocytogenes* populations decreased by 1.2- and 3.0-log within 24 h at 4°C, respectively. During the 28 d storage at 4°C, *L. monocytogenes* counts in untreated controls increased from the initial 4 log_10_ CFU/g to 8.3 log_10_ CFU/g. In the same condition, the overall growth of *L*. *monocytogenes* decreased by 0.3 to 2.6-log for cheese with 200 μg/ml of ELA and by 2.3 to 5.0-log for samples with 800 μg/ml of ELA. As reported by Woodcock et al. (2009), the aerobic plate count in unflavored milk with ELA (125, 170, or 200 μg/ml) remained below the regulatory limit of 20,000 CFU/ml for grade ‘A’ pasteurized milk during 21 days of storage at 6°C. However, the antimicrobial activity of ELA added to chocolate milk was reduced compared to unflavored milk, which might be due to the stabilizers (e.g., carrageenan) in the chocolate powder. The combinations of ELA with other antimicrobial agents were also used in dairy products such as essential oils (Ma et al., 2013), ε-polylysine, and nisin (Martínez-Ramos et al., 2020). According to Ma et al. (2013), the combination of ELA with cinnamon leaf oil or eugenol exhibited synergistic against *L*. *monocytogenes* in 2% reduced-fat milk. In the work of Martínez-Ramos et al. (2020), a synergistic interaction was observed for ELA and ε-polylysine against *L. monocytogenes* in queso fresco cheese.

### Aquatic products

Aquatic products, like any food item, can be contaminated with a variety of bacteria and viruses capable of causing disease in consumers (Iwamoto et al., 2010). The potential of ELA to improve the microbial safety and quality of aquatic products has been assessed in several studies (Soni et al., 2014; Zhuang et al., 2020). For instance, Soni et al. (2014) determined the efficacy of ELA for the inactivation of *L*. *monocytogenes* inoculated on cold-smoked salmon. The results indicated that the salmon treated with ELA (200 ppm) individually for 24 h at 4°C were able to achieve a 2.2 log reduction of *L*. *monocytogenes*. In recent work, Zhuang et al. (2020) assessed the effects of ELA on microbiota, quality, and biochemical changes of largemouth bass fillets during storage. The fillets were immersed in sterile water and 0.1% (w/v) ELA solution for 10 min, respectively, and then were stored at 4°C. The total viable counts (TVC) of ELA-treated samples were significantly lower than that of the control during the storage up to 11 d, suggesting that ELA was effective in the washing/cleaning of aquatic products. Meanwhile, ELA also attenuated effectively the changes in the color, TVB-N, ammonia concentration, and biogenic amines of chill-stored largemouth bass fillets. In addition, edible films incorporated with ELA were also used for aquatic products preservation. Demircan and Özdestan Ocak (2021) found that edible chitosan film coating with ELA (0.1%, w/v) significantly inhibits bacterial growth and biogenic amines formation of mackerel fillets during storage at 2°C. Similarly, active starch-gelatin films with ELA could effectively inhibit the microbial growth in marinated salmon and extend the product shelf life in terms of microbial spoilage (Moreno et al., 2017).

### Other food products

The antimicrobial effect of ELA has been investigated with other food products. Chen et al. (2015) investigated the antimicrobial activity of ELA against *Salmonella* in peanut paste at different fat concentrations. For peanut paste with different water activities (a_w_) of 1.0 and 0.7, 5,000 ppm of ELA reduced the population of *S.* Tennessee in low fat (< 5%) peanut paste by 0.92- and 4.08-log, respectively, after 5-day storage at 25°C. In addition, the counts of *S.* Tennessee in low fat (< 5%) peanut paste with a_w_ 0.5 and 0.3 were reduced to undetectable levels after 24 h with 5,000 ppm of ELA. The efficacy of ELA is also negatively affected by the fat concentration. For peanut paste with 50% fat, ELA at 5,000 ppm caused a 1.58-log reduction in 5 days compared with the control (Chen et al., 2015). In contrast, the spray application of 0.2% ELA and 200 ppm peracetic acid did not cause significant reductions in the aerobic plate count or *E. coli*/coliform counts of California walnuts (Frelka and Harris, 2015).

### Active food packaging

In recent decades, various antimicrobial substances have been wildly incorporated into food packaging systems to extend the shelf life of food products. Data from the literature indicate that ELA has been utilized to prepare active antimicrobial food packaging materials. Generally, ELA is incorporated into petroleum-based polymers (such as polyethylene and ethylene vinyl alcohol copolymers; Muriel-Galet et al., 2015; Manso et al., 2021) and biopolymers such as poly-γ-glutamic acid (Gamarra-Montes et al., 2018), polylactic acid, chitosan, pullulan, starch, and zein (Table 5). According to previous studies, ELA-incorporated food packaging materials exhibit excellent antimicrobial activity. For example, Muriel-Galet et al. (2015) prepared ethylene vinyl alcohol copolymers (EVOH) films containing ELA at 5% or 10% (w/w). All the ELA-incorporated films displayed antimicrobial capability against *L*. *monocytogenes* and *E*. *coli* both *in vitro* and in inoculated ready-to-eat surimi sticks. On the 10th day of storage at 4°C, *L*. *monocytogenes* and *E. coli* on samples wrapped in EVOH films with 10% ELA decreased by >3.25- and > 2.32- log, respectively.

**Table 5:** Applications of ELA-based antimicrobial films in food preservation.

Antimicrobial	Film substrate	Food product	Target microorganism	Antimicrobial activity	Reference
ELA (2.6%, w/w)	Polylactic acid	Cooked sliced ham	*L*. *monocytogenes* ATCC 19115 and *S*. Typhimurium DMST 0562	Bacterial populations decreased by 3.7log after the 7th day of storage at 4 °C	Theinsathid et al. (2012)
ELA (5%, w/w)	Chitosan and glycerol	Chicken breast fillets	Mesophiles, psychrophiles, *Pseudomonas* spp., colifoms, lactic acid bacteria, hydrogen sulfide-producing bacteria, yeast and fungi	1.78–5.81 log reduction during 8-day storage at 4 °C	Higueras et al. (2013)
ELA (50–200 μl/ml)	Chitosan	Ready-to-eat deli turkey meat	*L*. *innocua* ATCC 51742, 33,090, and 33,091	*L*. *innocua* on meat surface decreased by 1.8–2.4 log after 24 h incubation at 10°C	Guo et al. (2014)
ELA (5 and 10%, w/w)	Ethylene vinyl alcohol copolymers (EVOH)	Ready-to-eat surimi sticks	*L*. *monocytogenes* CECT 934 and *E*. *coli* CECT 434	After 10 days under storage at 4 °C, *L*. *monocytogenes* decreased by 1.77 to 3.25 log and *E*. *coli* decreased by 1.01 to 2.32 log	Muriel-Galet et al. (2015)
ELA (2%, w/v) and nisin Z (320 AU/ml)	Pullulan	Raw turkey breast, ham, raw beef slices	*Salmonella* spp. (ATCC 14028, ATCC 13331, and ATCC 10118), *L*. *monocytogenes* Scott A, *S*. *aureus*, *E*. *coli* spp. (O157:H7 ATCC 43895, O111, and O26)	*Salmonella* spp. on raw turkey breast slices decreased by 2.5 to 5.1 log; *S*. *aureus* and *L*. *monocytogenes* Scott A on ham surface decreased by 5.53 and 5.62 log, respectively; *E*. *coli* on raw beef slices: *E*. *coli* decreased by >4 log after film treatment and storage at 4 °C for 28 days	Pattanayaiying et al. (2015)
ELA (1.3%, w/w)	Oxidized corn starch, bovine gelatine and glycerol	Marinated salmon	Total viable counts (TVC) and *L*. *innocua* CECT 910	After 45 storage days at 5°C, TVC remained below the legal limit and *L*. *innocua* decreased by 0.98 log	Moreno et al. (2017)
ELA (10%, w/w)	Corn starch, bovine gelatin, and glycerol	Chicken breast	TVC, psychrotrophic bacteria (PB), lactic acid bacteria (LAB), anaerobic bacteria (AB), total coliforms (TC) and *E*. *coli*	The shelf life of chicken breast fillets was extended significantly (*p* < 0.05)	Moreno et al. (2018)
ELA (10%, w/w)	Zein, polypropylene and glycerol	Chicken soup	*L*. *monocytogenes* CECT 934 and *E*. *coli* CECT 434	*L*. *monocytogenes* and *E*. *coli* decreased by 3.21 and 3.07 log, respectively, after storage at 4 °C for 10 days	Kashiri et al. (2019)
ELA (2.5%)	Pullulan, gelatin, xanthan gum and glycerol	Raw beef	*E*. *coli*, *Salmonella* spp., *L*. *monocytogenes* and *S*. *aureus*	After storage at 4°C for 28 days, *E*. *coli*, *Salmonella* spp., *L*. *monocytogenes* and *S*. *aureus* onto raw beef slices decreased by 2.86, 3.04, 3.33 and 3.53 log, respectively	Hassan and Cutter, 2020
ELA (2%, w/w)	Chitosan	Frozen stored chicken drumsticks	Aerobic plate count (APC), psychrotrophs, *S*. *aureus*, and Enterobacteriaceae counts	All treated chicken drumsticks had a significant reduction in all investigated bacterial counts, pH, and thiobarbituric acid values and a significant improvement in sensory attributes.	Abdel-Naeem et al. (2021)
ELA (0.5, 1.0, 1.5, and 2.0%, w/v)	Polylactic acid	Srawberries	–	The active films with ELA effectively extended the shelf-life of strawberries at 25°C	Li et al. (2021)

Although there are various reports on the food packaging materials with ELA (Table 5), most of which are confined to the laboratory, due to the absence of suitable large-scale manufacturing processes for continuous production. The electrospinning process has been regarded as one suitable method for the large-scale production of long and continuous nanofibers. In several recent studies, the electrospinning technique was used to prepare polymer nanofibers with ELA. Deng et al. (2018) prepated chitosan/poly(ethylene oxide /ELA) composite nanofibrous films *via* electrospinning, which displayed excellent antimicrobial activity against *E*. *coli* and *Staphylococcus aureus*. In a recent work of Li et al. (2021), the authors prepared polylactic acid/ELA composite nanofibrous films *via* electrospinning. According to the results of the disc diffusion assay, the active films exhibited outstanding antimicrobial activity against *E*. *coli* O157:H7, *S*. *aureus*, and *Botrytis cinerea*. Meanwhile, the active films with ELA could also effectively extend the shelf-life of strawberries at 25°C. Summarily, considerable attention should be devoted to the large-scale production and practical application of active food packaging with ELA.

### Disinfection of food-contact surfaces

Food contact surfaces are considered to be the primary source of microbial contamination within food processing areas. Another potential application for ELA is the decontamination of food processing surfaces and equipment. Saini et al. (2013) investigated the efficacy of ELA against *L*. *monocytogenes* on polished stainless steel coupons. After exposure to ELA at 100 μg/ml for 5 and 10 min, *L. monocytogenes* with an initial level of 4 log_10_ CFU/coupon decreased by 1.38- and 2.57-log, respectively. Similar findings were observed in bacterial biofilms on food contact surfaces, such as stainless steel, rubber (Sadekuzzaman et al., 2017), and glass (Fu et al., 2017a; Fernández et al., 2018). As previously reported by Sadekuzzaman et al. (2017), the populations of *E*. *coli* O157:H7, *L*. *monocytogenes*, *S*. Enteritidis and *S*. Typhimurium in biofilms grown on stainless steel decreased from 6.0, 7.2, 5.4, and 5.1 log_10_ CFU/cm^2^ to values below the detection limit, respectively, after ELA treatment at 200 μg/ml for 2 h. Significant biofilm reduction in rubber surface was also observed for all the tested strains after ELA treatment (50–200 μg/ml) for 2 h.

## Combination of ELA with other technologies

Studies have indicated that ELA alone may not be sufficient for food preservation due to the influences of food constituents and treatment conditions (such as pH and temperature). The combination of ELA and other hurdles has been highlighted as a feasible strategy to enhance microbial inactivation by additive or synergistic effects. Some combined approaches have already proved successful in this regard, such as methylparaben (Li et al., 2018), nisin and ε-polylysine (Martínez-Ramos et al., 2020), essential oils or their constituents (Manrique et al., 2017), mild heat and ultraviolet (Yang et al., 2019), and high hydrostatic pressure (Seemeen, 2011).

### ELA with antibacterial agents

Several benefits have be resulted from the combination of ELA with other antibacterial agents, such as methylparaben (Li et al., 2018; Loeffler et al., 2020), ε-polylysine (Kozak et al., 2017; Martínez-Ramos et al., 2020), nisin (Pattanayaiying et al., 2014; Soni et al., 2014), potassium lactate and sodium diacetate (Martin et al., 2009), organic acid salts (Luchansky et al., 2005; Suksathit and Tangwatcharin, 2013), and peracetic acid (Shen et al., 2021).

Parabens, also known as para-hydroxybenzoic acid esters, are a class of antimicrobial preservatives allowed for use in foods. It is proven that ELA exhibits higher antibacterial and antifungal activities in combination with methylparaben (Li et al., 2018; Loeffler et al., 2020). As reported by Loeffler et al. (2020), the MIC values of ELA against *L*. *innocua* and *P*. *fluorescens* in nutrient broth with 2% bovine serum albumin (BSA) were 300 and 200 μg/ml, respectively. However, the MIC values of ELA against *L*. *innocua* or *P*. *fluorescens* decreased to 175 and 125 μg/ml, respectively, when combined with 0.1% methylparaben in the same testing condition. Similar findings were also obtained by Li et al. (2018) that ELA combined with methylparaben displayed enhanced antifungal activity. For instance, the MIC values of ELA and methylparaben against *P*. *italicum* were 400 and 800 μg/ml, respectively; while the MIC for ELA was reduced to 50 μg/ml in the presence of methylparaben at 200 μg/ml (Li et al., 2018).

ε-polylysine, a naturally antimicrobial cationic peptide, is commercially used as a safe food preservative globally. Martínez-Ramos et al. (2020) evaluated the antimicrobial combinations of ELA and ε-polylysine on *L. monocytogenes* growth in queso fresco. For the cheeses treated with ε-polylysine (250 μg/g) and ELA (200 μg/g), the *L. monocytogenes* population increased by approximately 1.5-log stored for 28 d at 4°C, lower than the control samples (~3 log_10_ CFU/g growth from the initial inoculum). Similar findings showed that the combined applications of ELA with ε-polylysine resulted in a significant reduction of *L. monocytogenes* in whole milk (Kozak et al., 2018) and *Salmonella* on sterile filter paper (Benli et al., 2011).

Nisin is a polypeptide bacteriocin and is commonly used as a food preservative. Pattanayaiying et al. (2014) investigated the effects of ELA and nisin, alone or in combination on the survival of bacteria. For instance, the population of *E*. *coli* O157:H7 decreased by 4.45-log after ELA treatment at 0.2 mg/ml for 6 h, while the bacterial cells were not inhibited by nisin at 320 AU/ml alone. Interestingly, the *E*. *coli* O157:H7 population decreased by approximately 7.16-log within the first hour of the combination treatment with ELA and nisin. Similarly, *L*. *monocytogenes* in cold-smoked salmon was reduced from 3.5 log CFU/cm^2^ to an undetectable level after the treatment of ELA (200 μg/ml) and nisin (500 μg/ml; Soni et al., 2014). The enhanced antimicrobial activity of ELA combined with nisin may be probably due to the formation of membrane channels and leakage of intracellular constituents such as potassium and phosphate ions (Pattanayaiying et al., 2014).

### ELA with essential oils

Essential oils are complex mixture of plant volatile compounds, which posse broad-spectrum antimicrobial activity. The antimicrobial activity of ELA combined with essential oils or their constituents was assessed in previous work (Ma et al., 2013, 2016a; Manrique et al., 2017). In a previous study, ELA (5 mg/l), cinnamon essential oil (200 mg/l), or EDTA (500 mg/l) did not exhibit antibacterial activity against *E*. *coli* O157:H7, *S*. *Enteritidis*, and *L*. *monocytogenes* in tryptic soy broth (TSB; Ma et al., 2016a). In contrast, the population of *E*. *coli* O157:H7, *S*. *Enteritidis*, and *L*. *monocytogenes* decreased by 4.70-, 5.01-, and 1.71-log, respectively, after the combined treatment of 5 mg/l ELA, 500 mg/l EDTA and 200 mg/l cinnamon essential oil (Ma et al., 2016a). Similarly, ELA combined with cinnamon essential oil exhibited much greater antibacterial activity against *L*. *monocytogenes* in 2% reduced-fat milk during 48 h of incubation at 21°C (Ma et al., 2013). Manrique et al. (2017) investigated the bacterial inactivation after the simultaneous or sequential application of ELA and eugenol. The simultaneous exposure of ELA and eugenol was found to be the most effective to inactivate *Staphylococcus carnosus* and *L*. *innocua*.

However, the application of essential oils as antibacterial agents is limited by their low water solubility, high volatility, and low long-term stability. The encapsulation of essential oils in nanoemulsions is an effective approach to overcome these limitations. As a cationic surfactant, ELA is widely used to prepare essential oil nanoemulsions. Chang et al. (2015) found that ELA addition substantially increased the antimicrobial efficacy of thyme oil nanoemulsions against *Zygosaccharomyces bailii*, an acid-resistant spoilage yeast. On the other hand, Ma et al. (2016b) reported that mixing ELA with lecithin could improve the physical properties of nanoemulsions based on thymol-eugenol mixtures. Nonetheless, the presence of nanosized lipid droplets in thyme oil-in-water nanoemulsions reduced the antifungal activity of ELA, which might be due to the partitioning of ELA between the lipid droplet surfaces and the yeast cell surfaces (Ziani et al., 2011). Therefore, future studies should concentrate on the interactions of surfactants and lipid droplets in essential oils nanoemulsions, which may help to improve the design of more effective antimicrobial delivery systems.

### ELA with mild heat

Recently, mild heat-based hurdles have been applied as novel food decontamination techniques. ELA combined with mild heating demonstrates synergistic antibacterial activity (Yang et al., 2019; Juneja et al., 2020). As reported by Yang et al. (2019), *E*. *coli* in sterile phosphate buffered saline (PBS) decreased by approximately 5-log after being treated with ELA (15 μg/ml) and mild heat (55°C) for 4 min. In contrast, ELA or mild heat alone demonstrated no significant effects on *E*. *coli* inactivation within 4 min of treatment. Significant synergistic inactivation of *L*. *innocua* was also observed after the combination treatment with ELA and mild heat under the same experimental condition.

Similar findings were also reported by Juneja et al. (2020), who examined the efficacy of ELA to reduce the *L*. *monocytogenes* population in ground beef following sous-vide processing at different temperatures. The D-values obtained from the Weibull model ranged from 43.74 to 4.47 min at 55–62.5°C. With the addition of ELA at 3 mg/g, the D-values at 55 to 62.5°C determined by the Weibull model were 22.71 and 1.60 min. ELA in beef increased the sensitivity of *L*. *monocytogenes* to sous-vide treatment, thereby extending the shelf-life and improving the product quality.

### ELA with non-thermal technologies

Non-thermal technologies, such as ultraviolet (UV) light, high hydrostatic pressure (HHP), ultrasound, cold plasma, and pulsed electric field, have been used in the sanitization of food products (Wu et al., 2020). These non-thermal technologies are operated at normal temperature conditions and with very short times, which helps to improve the sensorial and nutritional quality of foods. Several studies have reported the synergistic efficacy of ELA combined with non-thermal technologies, such as UV light (Yang et al., 2019) and HHP (Seemeen, 2011).

UV light possesses excellent germicidal properties against various microbial pathogens and has already been applied in the food industries. Yang et al. (2019) evaluated the antibacterial activity of ELA in combination with UVA. ELA treatment (15 μg/ml) alone inactivated 2 logs of *E*. *coli* O157:H7 in sterile PBS after 30 min of incubation, while UVA (320 to 400 nm) alone produced no significant inactivation of O157:H7 cells. In contrast, the *E*. *coli* O157:H7 population was decreased by approximately 6.5-log after the combined treatment with ELA and UVA for 30 min. A similar phenomenon was observed in *L*. *innocua* after ELA treatment alone or in combination with UVA. The authors also proposed that the synergistic action of ELA and UVA might be due to the enhanced oxidative stress and exacerbated membrane damage (Yang et al., 2019). HHP is an emerging non-thermal process technique and can inactivate harmful microorganisms in foods by intense pressure. Seemeen (2011) studied the effects of ELA and HPP on the shelf-life of ready-to-eat cooked chicken breast roast during storage at 4°C for 16 weeks. Aerobic plate counts (APCs) of chicken breast roast samples only decreased by 0.5- and 0.05-log, respectively, after exposure to ELA (200 μg/ml) or HHP (450 MPa for 1 min) alone followed by storage at 4°C for 16 weeks. For samples treated with HPP and ELA at 450 MPa for 1 min, APC decreased by 2.67-log. Therefore, ELA combined with HPP is an efficient method for extending the microbial shelf-life of the ready-to-eat sliced chicken breast roast.

### ELA with bacteriophages

Bacteriophages (phages) are bacterial viruses and can be used as narrow-spectrum antibacterials in food production for various advantages such as low inherent toxicity and no adverse environmental impact (Lewis and Hill, 2020). At present, some phage cocktails are available commercially and are currently used as either food additives or GRAS, such as ListShield^™^ and PhageGuard Listex^™^ P100. ELA does not affect the antibacterial activity of bacteriophages because phage particle is mainly composed of a protein molecule embedded in a capsid (Yang et al., 2017). The antibacterial activity of LEA combined with bacteriophages was investigated in several studies (Soni et al., 2012, 2014; Sukumaran et al., 2015; Yang et al., 2017).

According to previous studies (Yang et al., 2017), the counts of surviving *L*. *monocytogenes* on chicken breast decreased by 0.07- and 0.06-log, respectively, after a single round of treatment with bacteriophages (ListShield) or 100 mg/kg of ELA and storage at 4°C for 3 days. After the combined treatment with 100 mg/kg of ELA and bacteriophages, 0.43-log reduction was observed for *L*. *monocytogenes* on the chicken breast after storage at 4°C for 3 days. Moreover, the combined treatment with ELA and bacteriophage did not significantly affect the surface color parameters, sensory properties, pH, and thiobarbituric acid reactive substances (TBARS) content of chicken breasts (Yang et al., 2017). ELA combined with bacteriophages was also used for the preservation of queso fresco cheese (Soni et al., 2012) and cold-smoked salmon (Soni et al., 2014). As revealed by Soni et al. (2014), *L*. *monocytogenes* cells in cold-smoked salmon were reduced from 3.5 log_10_ CFU/cm^2^ to an undetectable level within 24 h after the combined treatments of ELA (200 ppm) with bacteriophage P100 (Listex^™^ P100, 10^8^ PFU/cm^2^). Further studies are still needed to reveal the complex mechanisms underlying combined treatment with ELA and bacteriophages.

## Factors affecting the antimicrobial activity of ELA

Though the antimicrobial of ELA has been already approved in previous publications, its effectiveness in practical application is still challenged by the treatment conditions and the natural specific characteristics of foods. Previous research shows that the antimicrobial efficacy of ELA is influenced by many factors, such as its concentration, exposure time, the particular properties of the microorganisms targeted, temperature, pH, and the characteristics of the treatment medium or foods.

### ELA concentration and treatment time

Generally, ELA exhibits enhanced antimicrobial activity at high concentrations. After ELA treatment at 0.001 and 0.01% with peracetic acid (80 ppm) for 30 s, *L*. *monocytogenes* with an initial population of 7.06 log_10_ CFU/mL decreased by 1.48- and more than 5- log reduction, respectively (Shen et al., 2021). The anti-biofilm potential of ELA is also significantly enhanced with increasing concentration (Sadekuzzaman et al., 2017; Fu et al., 2017a). ELA displays higher antimicrobial activity with increasing exposure time (Becerril et al., 2013; Shen et al., 2021). For instance, *L*. *innocua* in broth medium decreased by 2.5- and 4- log after exposure to 25 μg/ml of ELA for 2 and 4 min, respectively (Becerril et al., 2013). Similarly, *L*. *monocytogenes* incubated on apples decreased by 2.48- and 2.58-log after the combined treatment of ELA (0.05%) and peracetic acid (80 ppm) for 30 s and 2 min, respectively (Shen et al., 2021).

### Characteristics of microorganisms

The antimicrobial activity of ELA is influenced by the types, status, and population of microorganisms. As seen in Tables 1, 2, different microorganisms show various sensitivity to ELA. For instance, the MICs and MBCs of ELA against fungi are generally higher than that against bacteria, which may due to the different chemical composition and structures of their cell walls. Loeffler et al. (2014) assessed the antimicrobial efficacy of ELA against *S*. *cerevisiae*, *C*. *albicans*, and *Z*. *bailii*. *S*. *cerevisiae* was the most sensitive strain to ELA with a MIC value of 35 μg/ml (112.5 μg/ml for *C*. *albicans* and 62.5 μg/ml for *Z*. *bailii*). The antimicrobial efficacy of ELA is also affected by the serotypes of microorganisms. For instance, the MIC of ELA was 0.004% for *L*. *monocytogenes* 10403S (serotype 1/2a) and was 0.005% for *L*. *monocytogenes* 2045 (serotype 4b) at 37°C (Lingbeck et al., 2014). In addition, the specific physiological status of microbial cells (such as susceptibility and resistance, tolerance, persistence, and biofilm) may also affect the antimicrobial activity of ELA. For example, the antimicrobial activity of ELA is significantly affected by the biofilm growth age. After exposure to ELA at 80 μg/ml for 5 min, the viable bacterial of 2 h- and 24 h-old *E*. *coli* O157:H7 biofilms on cover glass decreased by 2.65- and 0.63- log, respectively (Fu et al., 2017a).

### Temperature

Temperature affects the antimicrobial activity of ELA. In the work of Lingbeck et al. (2014), *Listeria* and *Salmonella* were treated with ELA and then incubated at different temperatures for 24 h (4, 10, or 37°C for *Listeria* and 10, 25, or 37°C for *Salmonella*). The results showed that ELA exhibited stronger antibacterial activity when used at a higher incubation temperature. For example, the MICs of ELA against *Salmonella* were 0.072% at 10°C, 0.035% at 25°C, and 0.02% at 37°C, respectively. These results may be due to the changes in cellular structures and compositions of bacteria at different incubation temperatures, which further affect bacterial survival to environmental stresses (Cebrián et al., 2008). Similar findings were also reported by Taormina and Dorsa (2009b) that ELA was more effective to inactivate *L*. *monocytogenes* at 23°C (decreased more than 5.48 log) than at 4.4°C (decreased by 4.11 log) after only 5 min of exposure time. In addition, Yang et al. (2019) reported that ELA combined with mild heat (55°C) exhibited enhanced antibacterial activity against *E*. *coli* O157:H7 and *L*. *innocua*. Therefore, the combination of ELA and mild heat represents a promising strategy to eliminate microorganisms in foods. Finally, it should also point out that high temperatures may accelerate the hydrolysis of ELA. So ELA cannot be used at too high temperatures.

### pH

ELA has been shown to maintain antimicrobial activity over a wide pH range from 3 to 7, which may be used as an antibacterial agent for a wide range of food products. However, low or high pH may result in more extensive hydrolysis of ELA, thereby resulting in a decrease in its antibacterial activity. According to previous studies, ELA is easily decomposed under basic conditions. The half-life of ELA is greater than 1 year at pH 4, 57 d at pH 7, and 34 h at pH 9 during 25°C storage (EFSA, 2007), suggesting its decomposition by base-catalyzed hydrolysis. Therefore, special attention should be paid to the pH of food products for the practical use of ELA.

### Food matrices and components

ELA may interact with other components within foods and beverages, such as starch (Ma et al., 2013), proteins (Loeffler et al., 2020), polysaccharides (Loeffler et al., 2014), and lipids (Ziani et al., 2011; Magrinyà et al., 2015), causing a significant decrease in its antimicrobial activity. In general, many studies confirm that the concentration of ELA required to inactivate microorganisms in foods is higher than that needed for *in vitro* tests. Ma et al. (2013) reported that ELA effectively inhibited *L*. *monocytogenes* in TSB with a MIC of 11.8 μg/ml. However, potato starch at 2–5% (w/v) in TSB increased the MIC of ELA to 93.8–187.5 μg/ml. In addition, *L*. *monocytogenes* in 2% reduced-fat milk were only decreased by 1.02-log from the initial count of 7.31 log_10_ CFU/mL after incubation with 375 μg/ml of ELA at 32°C for 24 h (Ma et al., 2013). Similarly, the MIC values of ELA against *L*. *innocua* and *P*. *fluorescens* in nutrient broth (NB) were remarkably increased by 4–13 fold in the presence of BSA, whey protein isolate, or soy protein hydrolysate (Loeffler et al., 2020). Some additives used in foods and beverages also may affect the antimicrobial activity of ELA. In the work of Loeffler et al. (2014), the authors investigated the antimicrobial efficacy of ELA in the presence of xanthan and λ-carrageenan, two anionic polysaccharides used widely in beverages. The MIC values of ELA against *S*. *cerevisiae*, *C*. *albicans*, and *Z*. *bailii* were increased significantly with the increasing polysaccharide concentration (Loeffler et al., 2014). Chen et al. (2015) found that the fat concentration of peanut paste negatively impacted the antimicrobial efficacy of ELA. Magrinyà et al. (2015) also revealed that the antimicrobial efficacy of ELA was decreased with increasing fat addition (0 to 15 wt.%). These results might be due to that more ELA was present at the interface between water and fat, therefore, leading to the decreased antimicrobial effect. However, when the fat content increased from 15 to 50 wt.%, more ELA might again be present in the aqueous phase, causing a dramatic increase in the antimicrobial activity of ELA (Magrinyà et al., 2015).

Food matrices and components may protect microorganisms from the washing or disinfection treatment of ELA. In addition, ELA can form complexes with some charged food components (such as proteins and polysaccharides) by electrostatic interaction (Loeffler et al., 2014, 2020). Food ingredients with no charge, e.g., starch, may reduce the antimicrobial activity of ELA by increasing the viscosity, thereby limiting access of ELA to microorganisms cells (Ma et al., 2013). Furthermore, foods are characterized by a higher amount of available nutrients compared to *in vitro* assays, which may enable microorganisms to repair cellular damage and maintain homeostasis, leading to a decreased sensitivity if exposed to ELA.

## Conclusions and future perspectives

ELA exerts strong antimicrobial activity against a wide range of food pathogens and spoilage microorganisms. The antimicrobial action of ELA may be mainly attributed to its amphiphilic structure, membrane damage, and oxidative stress. As a promising antimicrobial agent, ELA has been widely exploited to improve the safety and quality of foods. Nevertheless, more and further studies are still required. The antibacterial mechanisms of ELA should be further elucidated with multi-omics techniques and molecular dynamics simulation. The lower antimicrobial efficacy of ELA is observed when in foods and beverages. Thus, much more work is needed to understand the interactions between ELA and the various components of foods. Also, more attention should be paid to the combination of ELA with other existing antimicrobials or technologies in food processing to improve the antibacterial efficacy of ELA. ELA-based antimicrobial packaging has been recognized as a promising form of active food packaging and an emerging technology, while more special attentions should be devoted to its commercial application.

## Author contributions

YfM and YqM: writing – review and editing. DZ and SW: writing original – draft. LC: review and editing. QX: conceptualization and supervision. All authors reviewed the manuscript, contributed to the article, and approved the submitted version.

## Funding

The work is financially supported by the Natural Science Foundation of Henan Province (No. 212300410090) and the Collaborative Innovation Special Project of Zhengzhou (No. 2021ZDPY0201).

## Conflict of interest

The authors declare that the research was conducted in the absence of any commercial or financial relationships that could be construed as a potential conflict of interest.

## Publisher’s note

All claims expressed in this article are solely those of the authors and do not necessarily represent those of their affiliated organizations, or those of the publisher, the editors and the reviewers. Any product that may be evaluated in this article, or claim that may be made by its manufacturer, is not guaranteed or endorsed by the publisher.
