# Corrigendum: Ethyl lauroyl arginate: an update on the antimicrobial potential and application in the food systems industry: a review

**DOI:** 10.3389/fmicb.2023.1216552

**Published:** 2023-06-26

**Authors:** Yunfang Ma, Yanqing Ma, Lei Chi, Shaodan Wang, Dianhe Zhang, Qisen Xiang

**Affiliations:** ^1^College of Food and Bioengineering, Zhengzhou University of Light Industry, Zhengzhou, China; ^2^Henan Key Laboratory of Cold Chain Food Quality and Safety Control, Zhengzhou, China

**Keywords:** ethyl lauroyl arginate, antimicrobial efficacy, decontamination, food, mechanism

In the published article, there was an error in the name and abbreviation of the chemical referred to as “lauric arginate ethyl ester,” abbreviated to “LAE.” It should be “ethyl lauroyl arginate” and abbreviated to “ELA.”

Owing to this error, the article title is corrected from “Lauric arginate ethyl ester: an update on the antimicrobial potential and application in the food systems” to “Ethyl lauroyl arginate: an update on the antimicrobial potential and application in the food systems industry: a review.”

In the abstract and throughout the text of the article, “lauric arginate ethyl ester” has been corrected to “ethyl lauroyl arginate,” and the abbreviation “LAE” has been corrected to “ELA.”

The keyword “lauric arginate ethyl ester” has been corrected to “ethyl lauroyl arginate.”

There are errors in the legends for Figure 1, Figure 2, Figure 3, Figure 4, Table 1, Table 2, Table 3, Table 4, and Table 5. The correct legends appear below.

Figure 1. Chemical structure of ELA.

Figure 2. Proposed metabolic pathway of ELA (EFSA, 2007; Hawkins et al., 2009).

Figure 3. Proposed mechanisms underlying the antimicrobial action of ELA. ROS, reactive oxygen species.

Figure 4. The application of ELA in the food industry.

Table 1. MICs and MBCs values of ELA against bacteria.

Table 2. MICs and MBCs values of ELA against yeasts and fungi.

Table 3. Effects of ELA on microbial inactivation meat and meat products.

Table 4. Effects of ELA on microbial inactivation in fruits and vegetables.

Table 5. Applications of ELA-based antimicrobial films in food preservation.

In the published article, there was an error in Figure 3 as published. “LAE” should be corrected to “ELA”. The corrected Figure 3 appears below.

**Figure 3:**
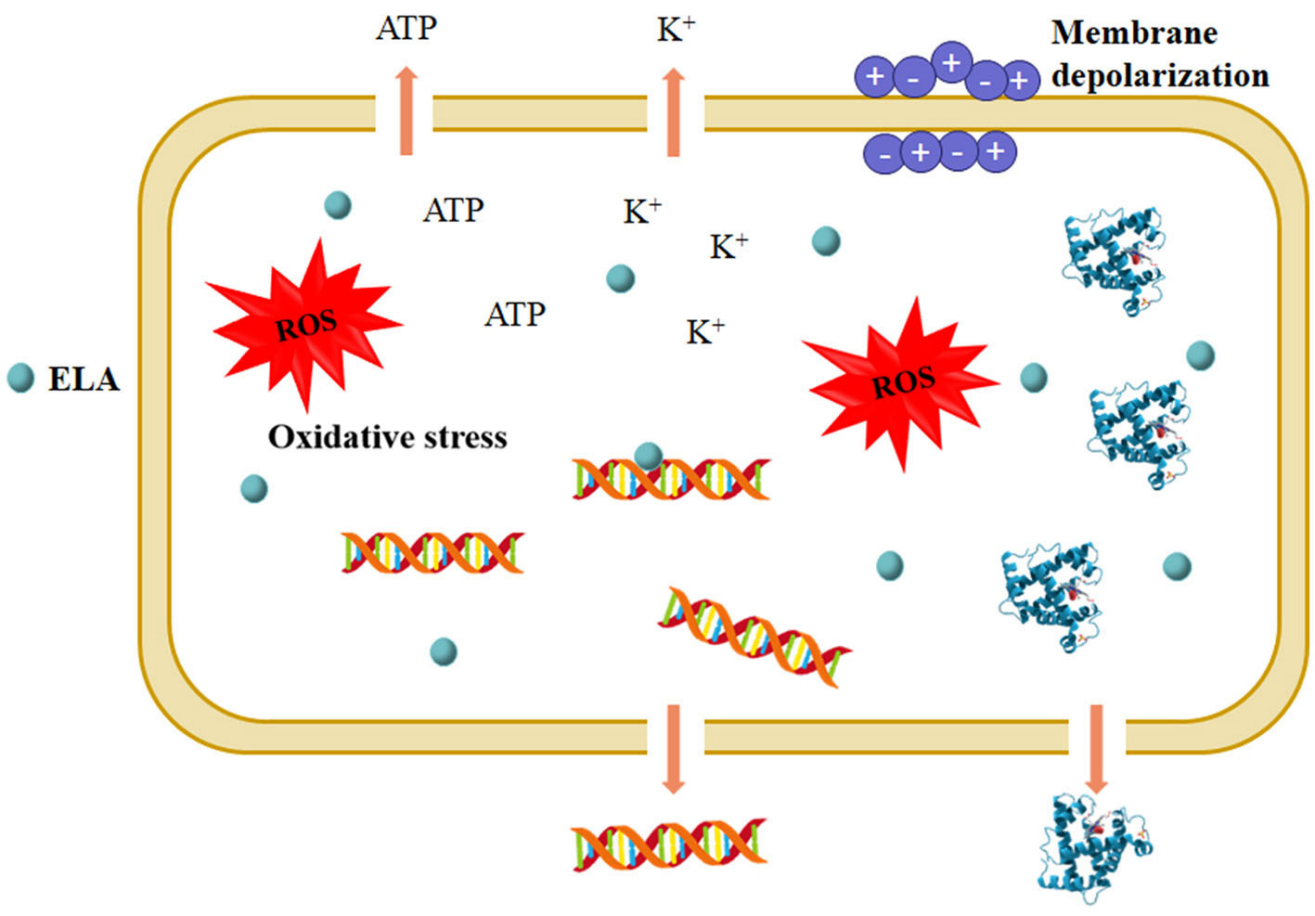
Proposed mechanisms underlying the antimicrobial action of ELA. ROS, reactive oxygen species.

The authors apologize for these errors and state that these do not change the scientific conclusions of the article in any way. The original article has been updated.

